# Comprehensive microarray analysis for the identification of therapeutic targets within HIF-1α signalling networks in diet-induced obesity via hypothalamic inflammation

**DOI:** 10.20945/2359-4292-2024-0098

**Published:** 2025-02-28

**Authors:** Hai Guo, Lijuan Ma, Dilihumaier Duolikun, Qiaoling Yao

**Affiliations:** 1 Department of Anesthesiology, the First Affiliated Hospital of Xinjiang Medical University, Urumqi, Xinjiang, China; 2 Department of Physiology, School of Basic Medical Sciences, Xinjiang Medical University, Urumqi, Xinjiang, China; 3 Xinjiang Perioperative Organ Protection Laboratory (XJDX1411), Urumqi, Xinjiang, China; 4 Xinjiang Key Laboratory of Molecular Biology for Endemic Diseases, Urumqi, Xinjiang, China; 5 State Key Laboratory of Pathogenesis, Prevention and Treatment of High Incidence Diseases in Central Asia, Urumqi, Xinjiang, China

**Keywords:** Diet-induced obesity, hypoxia-inducible factor (HIF)-1α, immune infiltration, hypothalamus, ksr2

## Abstract

**Objective:**

A high-fat diet (HFD) significantly contributes to obesity and alters the neurological function of the brain. This study explored the influence of hypoxia-inducible factor (HIF-1) and its downstream molecules on obesity progression in the context of HFD-induced hypothalamic inflammation.

**Materials and methods:**

Utilizing a bioinformatics approach alongside animal models, targets and pathways related to hypothalamic obesity were identified via network analysis, gene target identification, gene ontology analysis, Kyoto Encyclopedia of Genes and Genomes (KEGG) pathway enrichment, and subsequent validation in animal models.

**Results:**

HIF-1α has the potential to regulate the immune response by promoting immune infiltration and increasing the population of immune cells, particularly memory CD4 T cells, in the hypothalamus, primarily through its influence on ksr2 expression. Additionally, the analysis predicted five drugs capable of enhancing HIF-1-Ksr2 signalling.

**Conclusion:**

In conclusion, targeting Ksr2 with specific drugs represents a potential approach for addressing HFD-induced obesity. These novel findings lay the groundwork for developing dietary supplements and therapeutic interventions.

## INTRODUCTION

A high-fat diet (HFD) significantly contributes to obesity and alters neurological function (^1^). According to previous studies, a high-fat and high-sucrose diet can affect the hypothalamus to regulate the energy balance of the human body (^1^-^3^). These factors include hormone production, body temperature regulation, and blood glucose concentration (^4^,^5^). A previous study also suggested that dietary obesity was associated with an atypical form of proinflammatory signalling activation leading to background-level inflammation in the hypothalamus (^6^). This hypothalamus microinflammation may even have the potential to affect the ageing process (^7^-^9^). The intricate role of the hypothalamus in maintaining energy balance offers a crucial foundation for addressing obesity.

One potential mechanism underlying HFD-induced hypothalamic inflammation is the presence of saturated fatty acids (SFAs), which can trigger the activation of hypothalamic glial cells, leading to the accumulation of cytokines such as interleukin 1β (IL-1β) and tumour necrosis factor α (TNF-α). This cascade ultimately leads to an inflammatory response (^10^,^11^). Another previous study highlighted the ability of hypoxia to increase cholesterol and induce lipid peroxidation, independent of obesity (^12^). Additionally, the severity of hypoxia determines the degree of metabolic disruption. The reliance of neuronal metabolism on oxygen sensing illustrates how different oxygen levels create diverse metabolic states (^13^).

Hypoxia-inducible factor (HIF), a dimeric protein composed of a short-life α-subunit and a b constitutively expressed subunit, is the major transcription factor that regulates gene expression in response to hypoxia by inducing or suppressing genes (^14^). HIF-a is classified into three subtypes: HIF-1a, HIF-2a, and HIF-3a (^15^,^16^). HIF-1a plays a critical role in weight regulation, liver metabolism, cardiac metabolism, cancer metabolism, amino acid metabolism, and glucose homeostasis (^17^-^21^). Recently, some studies on appetite control in the brain have shown that HIF-1α is highly expressed in the hypothalamus in mice with morbid obesity (^22^). Some obesity-related genes regulated by HIF-1α also regulate energy metabolism (^19^). Furthermore, HIF-1α expression and stability increase in the hypothalamus of a HFD-induced obese mouse model (^19^).

Therefore, the aim of this study was to explore how HIF-1 and its downstream components contribute to obesity amidst hypothalamic inflammation caused by a HFD. We also aimed to propose potential drugs targeting HIF pathways and associated genes to combat HFD-induced obesity.

## METHODS

### Ethics statement

This study was performed in compliance with the Ethics Guidelines of Animal Fairwell and Care and approved by the institutional animal care review committee of the Animal Experimental Ethical Inspection Committee of the Laboratory Animal Centre, Xinjiang Medical University (No. 2015004). This study followed the Guide for the Care and Use of Laboratory Animals.

### Microarray data

GEO, a public genomics repository (http://www.ncbi.nlm.nih.gov/geo), provides extensive gene expression data through microarrays (^23^). The series matrix files and data table header descriptions of GSE127056 (^24^) and GSE104709 (^25^) in the GEO were downloaded to screen and validate genes expressed in the mouse hypothalamus. The GSE127056 dataset, which utilizes the GPL6246 (Affymetrix Mouse Gene 1.0 ST Array) platform, encompasses six samples from HFD-fed mice and three from those fed a normal diet, all from the hypothalamus. The GSE104709 platform, which uses the GPL21103 platform (Illumina HiSeq 4000 for *Mus musculus*), consists of five samples from mice fed a HFD and five from those fed a normal diet. The Combat algorithm of the SVA package (^26^) in R software (version 4.1.2) was used to eliminate chip batch effects, enabling the exploration of distinct molecular mechanisms between the sample groups. The limma package (^27^) in R software was used to detect genes with differential expression between the control on a normal diet and the HFD-fed groups, employing a significance threshold of P < 0.05 for screening. R’s heatmap package generated the heatmap for differentially expressed genes, whereas the ggplot2 package facilitated volcano plot analysis via the same software.

### Functional analysis of differentially expressed genes

Gene Ontology (GO) and Kyoto Encyclopedia of Genes and Genomes (KEGG) analyses were used to assess the related available categories. The significant categories included GO- and KEGG-enriched pathways with p values and q values < 0.05. The ssGSEA algorithm (^28^) was used to quantify the metabolic levels of the DEGs across all the samples, visualizing metabolic pathways through a heatmap.

### WGCNA

The R WGCNA package was used to explore coexpression relationships among differentially expressed genes (^29^). We used the WGCNA-R package, with a soft threshold of 10, to construct coexpression networks of the DEGs. This method converts the weighted adjacency matrix to a topological overlap matrix (TOM), estimating network connections. Hierarchical clustering was used to identify gene modules displayed in distinct colours, organizing genes by shared expression patterns and revealing their interactions.

### Immune microenvironment network analysis

CIBERSORT (^30^) employs support vector regression to deconvolve immune cell subtypes from an expression matrix. It includes 547 biomarkers distinguishing 25 phenotypes of murine immune cells, encompassing T, B, plasma, and myeloid subsets. CIBERSORT was used to analyse the sample data, inferring the relative proportions of 25 immune-infiltrating cells. A Pearson correlation analysis between gene expression and immune cell content was conducted, and the results were visualized via ggplot2.

### GSEA analyses

Gene set enrichment analysis (GSEA; Broad Institute, Inc., Massachusetts Institute of Technology, and University of California Reagents) was used to detect significant expression differences within defined gene sets between two groups (^31^). The database for annotation, visualization, and integrated discovery [DAVID 6.8 http://david.ncifcrf.gov; (^32^,^33^)], an online bioinformatics resource, systematically annotates genes and proteins via diverse biological data. GSEA was used to examine signalling pathways among the highand low-HIF-1-related gene groups, revealing potential molecular variances. The analysis ranked genes and evaluated enrichment within predefined sets, elucidating molecular differences between sample groups, using a limit of 1,000 substitutions and phenotype definitions.

### Transcription regulation analysis

CistromeDB (^34^) is a vast repository housing 30,451 human and 26,013 mouse ChIP-seq and DNase-seq samples. We explored the regulatory links between transcription factors and key genes via the Cistrome DB, which aligns with mm10 and a 10-kb range around the transcription start site. Visualization of the data was performed via Cytoscape (^35^).

### Animal experiments

Male HIF-1α^flox/flox^ mice bred at Xinjiang Medical University from the Polotsky Laboratory stock (Johns Hopkins University) were housed under controlled SPF-grade conditions. After surgical viral injections with AAV-hSyn-GFP and AAV-hSyn-cre-GFP viruses into the hypothalamic base, the mice were split into two groups: control (AAV-hSyn-GFP) and HIF1αKOMBH (AAV-hSyn-cre-GFP). Daily monitoring of body weight and food intake began on the 5th day postinjection and continued until the 28th day, which was the fourth week after virus administration.

### Energy metabolism and behaviour monitoring of the mice

A Promethion animal metabolism and behaviour monitoring system (Sable Systems International, USA) was utilized to measure energy metabolism-related parameters in the mice. This system enables real-time monitoring of mouse energy expenditure and can measure several key indicators, including mouse activity level, oxygen consumption (VO_2_), carbon dioxide production (VCO_2_), and energy expenditure (EE). The formula for calculating EE is as follows: 3.941 × VO_2_ (litres/day) + 1.106 × VCO_2_ (litres/day).

The monitoring procedure was as follows: control and HIF1αKOMBH mice 30 days after viral infection were separately placed in individual monitoring chambers equipped with bedding material, where 50 g of food and 100 mL of drinking water were provided. The first 48 hours after the mice were placed in the monitoring chambers were designated the adaptation phase, during which no data collection occurred. After the adaptation period, the system began automatically collecting data on mouse activity, VO_2_, and other parameters every 5 minutes, which were continuously recorded for 24 hours. The metabolic chambers were maintained under standard light-dark cycles (lights on at 8:00 AM, off at 8:00 PM) and at a constant temperature of 24 °C.

### Histological staining of mouse epididymal fat and scapular brown adipose tissue

The mice were fasted for 6 hours, anaesthetized, and rapidly perfused with sterile physiological saline and 4% paraformaldehyde four weeks after virus injection. Brown adipose tissue from the scapular region and epididymal fat tissue (0.5*0.5 cm) were collected. The tissue blocks were removed from the fixation solution and washed twice with PBS. The fixed tissue blocks underwent gradient ethanol dehydration, transparency, embedding in paraffin, paraffin section preparation, deparaffinization, haematoxylin, and eosin (HE) staining, and sealing on slides with neutral mounting medium. A light microscope and image acquisition system were used to observe and record the morphology of the adipose tissue cells. ImageJ was used to calculate the average cell size in the captured slide images.

### Gene expression patterns in the hypothalamus

Hypothalamus tissue was collected and processed for gene expression analysis. RNA was extracted via TRIzol^®^ reagent, reverse-transcribed into cDNA via random primers, and then assessed for selected gene expression via qPCR via designed primers from Oebiotech (Shanghai, China).

### CMAP analysis

The CMAP database of the Broad Institute links genes, small molecules, and diseases via gene expression. It encompasses microarray data before and after treatment with 1309 small-molecule drugs across various cancers, aiding in predicting obesity-specific targeted therapy drugs based on differentially expressed obesity genes.

### Statistical analysis

Statistical analysis was performed via the R language (version 4.1.2). All the statistical tests were two-sided, and p < 0.05 was considered statistically significant.

## RESULTS

### Differential gene expression in the hypothalamus of HFD-fed mice

**Figure 1:**
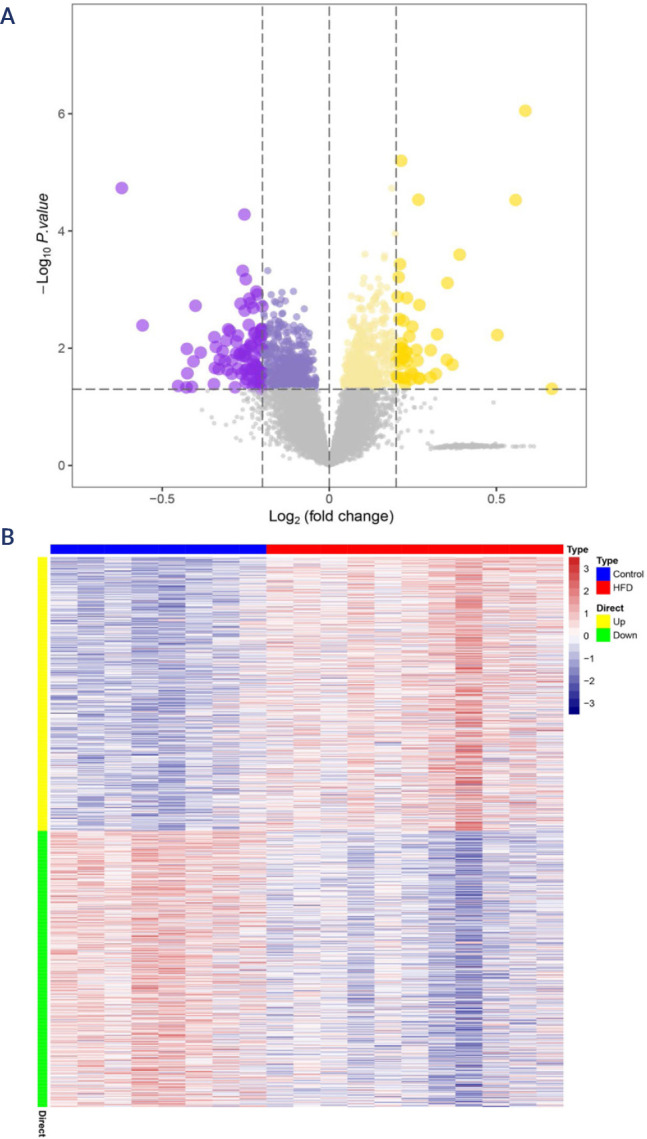
Genome-wide analysis of the hypothalamus of HFD-fed mice. The series matrix files of GSE127056 and GSE104709 were downloaded from the GEO database to screen and verify genes expressed in the mouse hypothalamus. **(A)** Volcano plot analysis was used to identify differentially expressed genes. The yellow and purple dots represent upregulated and downregulated genes, respectively, in the hypothalamus tissue from the HFD groups compared with the normal controls. **(B)** Heatmap of 1173 differentially expressed genes screened by the limma package. Red areas represent highly expressed genes, and blue areas represent genes expressed at low levels in hypothalamus tissue from HFD groups compared with normal controls. HFD: high-fat diet.

**Supplementary Figure 1:**
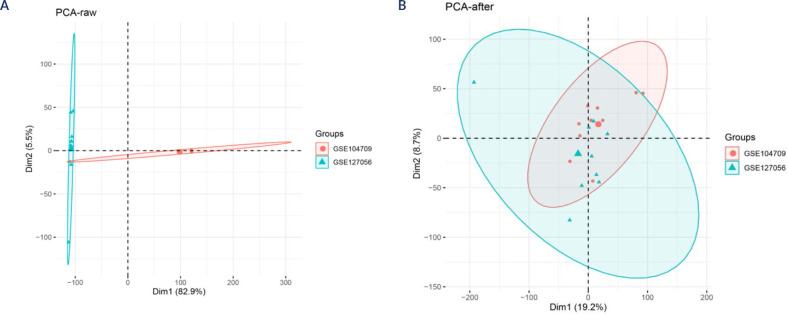
The batch effect between chips is reduced after Combat algorithm correction. A plot of principal component analysis (PCA) factors related to obesity was generated from the GEO datasets GSE127056 and GSE104709. (**A**) Before combat algorithm correction. (**B**) After combat algorithm correction.

We analysed the GEO datasets GSE127056 and GSE104709 to identify obesity-related differentially expressed genes in the hypothalamus of HFD-fed mice. The dataset included 19 samples (control group, n = 8; HFD group, n = 11). Using the Combat algorithm, chip correction reduced batch effects **(Supplementary Figure 1)**. After consolidating and normalizing the data, Limma identified 1173 DEGs (584 upregulated and 589 downregulated) between the groups **(Figure 1A and B)** under screening conditions of p < 0.05.

**Figure 2:**
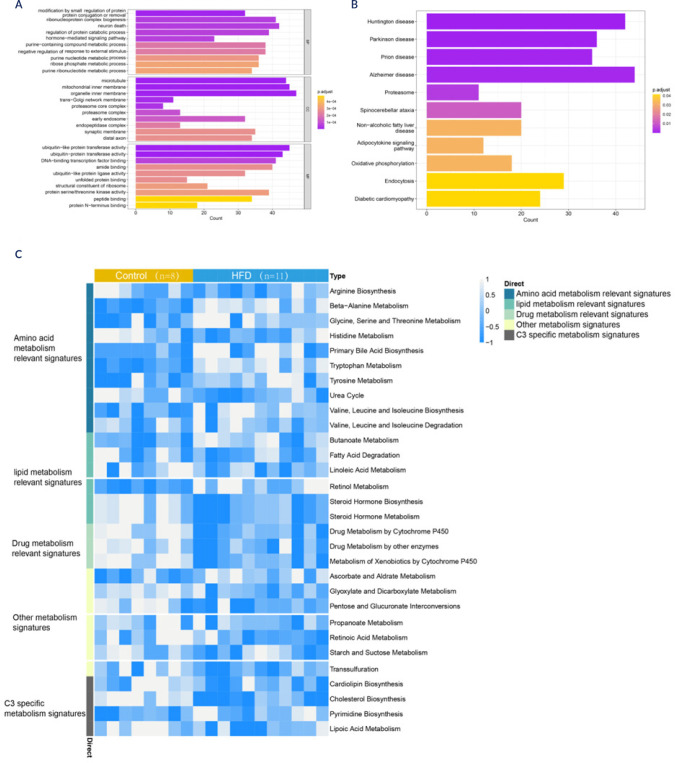
GO and KEGG pathway enrichment analyses of the hypothalamus of HFD-fed mice. The series matrix files of GSE127056 and GSE104709 were downloaded from the GEO database. **(A)** Top 10 gene ontology (GO) terms associated with the biological process (BP), cellular component (CC), and molecular function (MF) categories. **(B)** Target KEGG pathway network of differentially expressed genes. **(C)** Heatmap of metabolic pathway enrichment analysis.

We conducted enrichment analysis on the DEGs to explore their coexpression relationships. Gene Ontology (GO) enrichment revealed pathways related to protein catabolic processes, purine metabolism, ribose phosphate metabolism, and proteasome functions **(Figures 2A)**. KEGG enrichment highlighted pathways such as oxidative phosphorylation and adipocytokine signalling, among others **(Figure 2B)**. Additionally, the metabolic pathway heatmap revealed elevated scores for amino acid metabolism in the HFD samples but lower scores for drug metabolism and other metabolic categories than in the normal samples **(Figure 2C)**.

### Gene sets regulated by HIF-1α in the hypothalamus of HFD-fed mice

**Figure 3:**
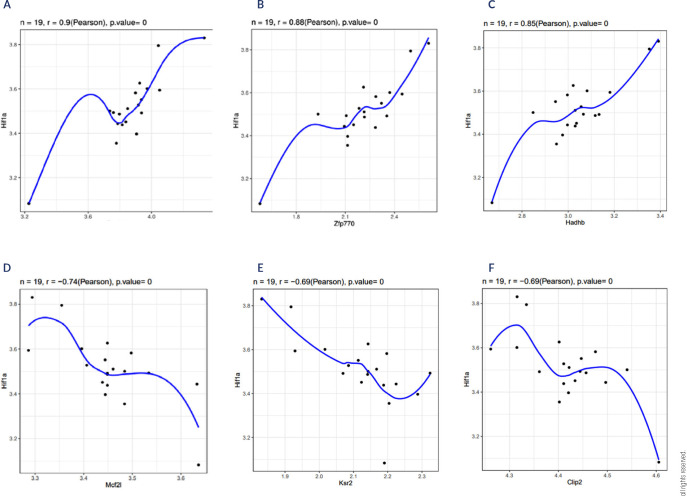
HIF-1α-related hub targets affecting obesity in the hypothalamus of HFD-fed mice. The WGCNA package in R software was used to investigate the coexpression relationship between HIF-1 and differentially expressed genes. **(A)** Dyntl3; **(B)** Zfp770; **(C)** Hadhb; **(D)** Mcf2l; **(E)** Ksr2; **(F)** Clip2.

**Supplementary Figure 2:**
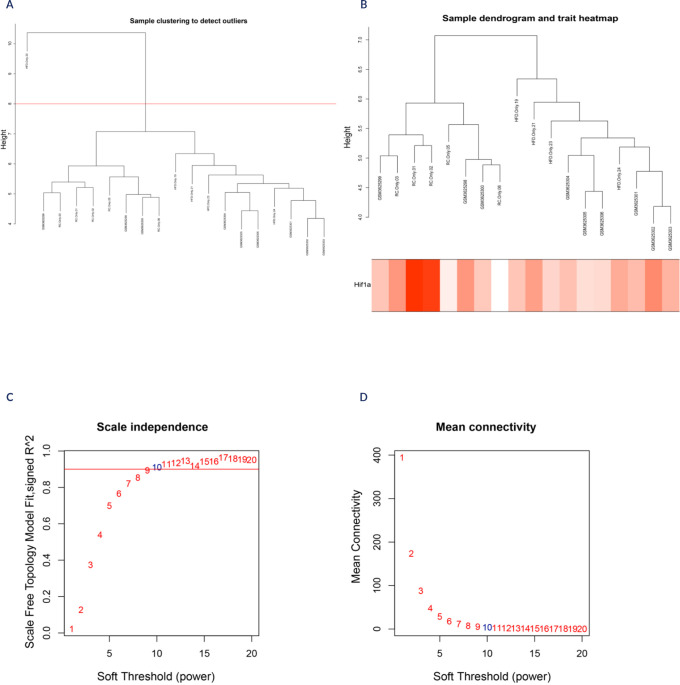
The WGCNA network is based on the expression data of differentially expressed genes and was used to explore the HIF-1α-related coexpression network. **(A)** Cluster dendrogram. The red line represents the cut-off of data filtering in the data processing step. **(B)** Sample dendrogram and trait indicators. **(C)** Analysis of the scale-free fit index for various soft-thresholding powers. The red line represents the cut-off value. **(D)** Analysis of the mean connectivity for different soft thresholding powers.

**Supplementary Figure 3:**
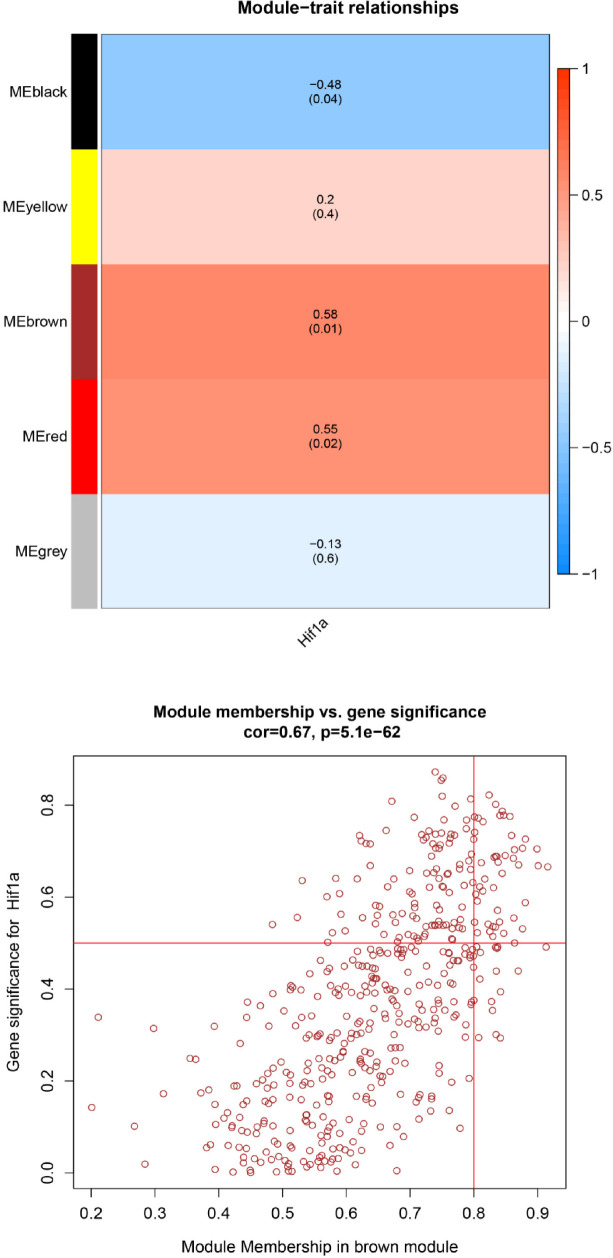
Main findings in the module-trait correlation analyses. Heatmap of the correlations between modules and HIF-1α (each gene module contained a correlation coefficient and corresponding p value). The gene significance for HIF-1α in the turquoise module (one dot represents one gene in the turquoise module).

We used expression data of differentially expressed genes to construct a WGCNA network, setting the shear height to 8 and the soft threshold to 10 **(Supplementary Figure 2)**. Five gene modules were identified: black (403), yellow (116), brown (466), red (85), and grey (103). An analysis of the relationships between the modules and traits revealed that the brown module was highly correlated with HIF-1α (cor = 0.58, p = 0.01) **(Supplementary Figure 3)**. To identify genes strongly correlated with HIF-1α in the brown module, we extracted genes with |GS|>0.5 and |MM|>0.8 for further analysis; this revealed six hub genes: Dynlt3, Zfp770, Hadhb, Mcf2l, Clip2, and Ksr2 **(Figure 3)**.

### Immune cell expression profile in the hypothalamus of HFD-fed mice

**Figure 4:**
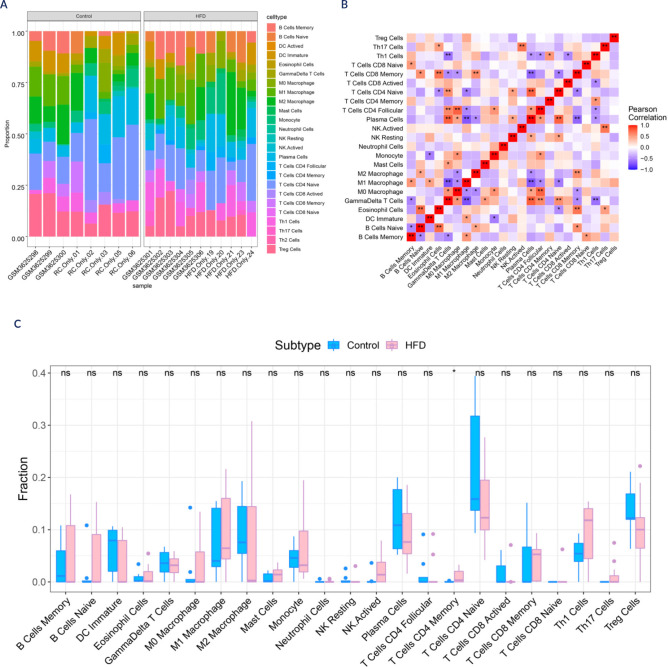
Relationships between the 6 hub genes and immune cell infiltrates in the hypothalamus of HFD-fed mice. The correlation between hub gene expression and resistant cell content was visualized via ggplot2. **(A)** Immune cells contributing to the HFD and control samples. **(B)** Correlations among immune cells. **(C)** The difference in the number of resistant cells between the HFD and control groups. HFD: high-fat diet.

**Supplementary Figure 4:**
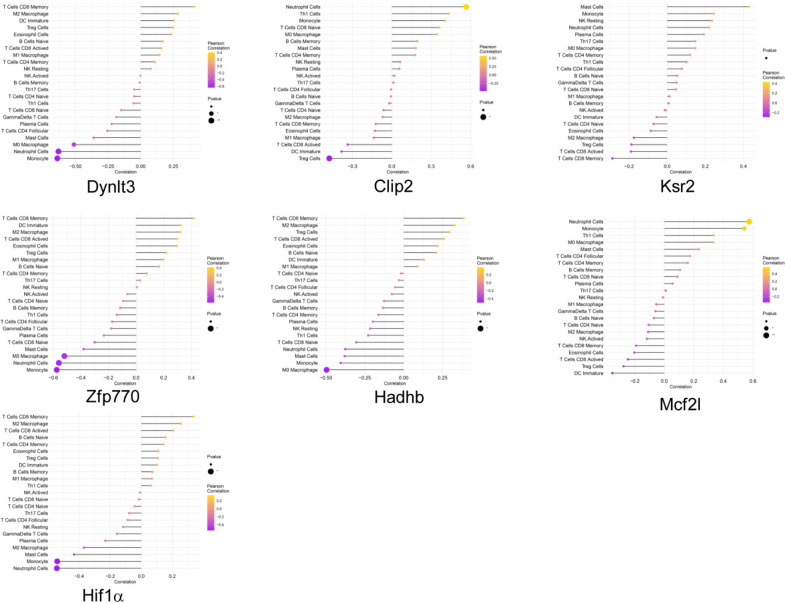
Correlations between the 7 genes and immune cells. Correlation matrix of the Pearson correlation coefficients.

The microenvironment, including immune cells, impacts disease diagnosis and treatment sensitivity. To analyse the role of the hub genes in obesity, we studied their relationship with immune infiltration. **Figures 4A and 4B** display the immune cell proportions and correlations. The number of memory T CD4+ cells notably increased in the HFD-fed samples **(Figure 4C)**. The six genes strongly correlated with the immune cell content **(Supplementary Figure 4)**. Dynlt3 and Zfp770 were negatively correlated with M0 macrophages, neutrophils, and monocytes; Hadhb was inversely correlated with M0 macrophages; Mcf2l was positively correlated with neutrophils and monocytes; and Clip2 was positively correlated with neutrophils. Additionally, HIF-1α was negatively correlated with monocytes and neutrophils, indicating that immune cell infiltration is influenced by these genes.

### HIF-1α^flox/flox^ mice showed signs of obesity

**Figure 5:**
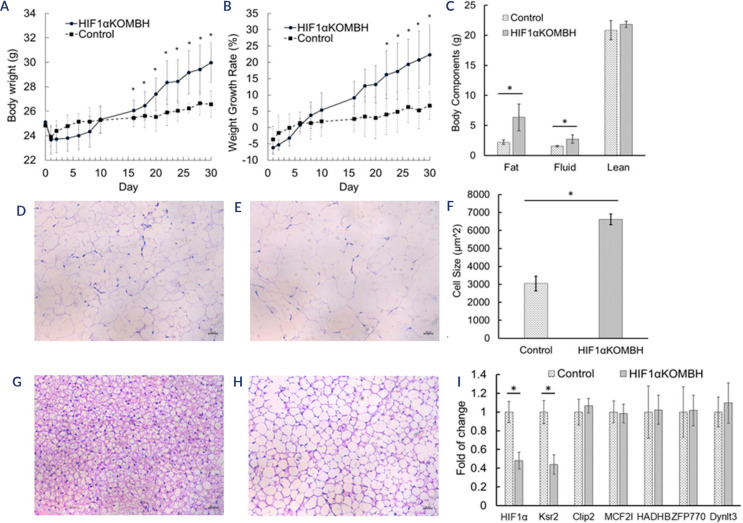
Animal model of HIF-1a ^flox/flox^ used in the search for results. In the HIF1aKOMBH group, significant differences were found in the body weights 16 days after virus injection **(B)** and in the weight gain rate 22 days after virus injection **(C)**. The body composition also differed between the two groups **(D)**. The energy efficiency of the HIF1aKOMBH group was significantly lower than that of the control group **(E)**. HE staining of epididymal adipose tissue revealed that the number of cells in the HIF1aKOMBH group **(G)** was significantly greater **(H)** than that in the control group **(F)**. The same phenomena can also be observed in brown adipose tissue **(I-K)**. The qPCR results revealed that the ksr2 gene was the only selected gene underregulated in the hypothalamus of the HIF1aKOMBH group. The scale bars in the images represent 25 µm, and * represents a significant difference **(L)**.

Compared with control mice, HIF1αKOMBH mice consumed more food 10 days after virus injection **(Figure 5A)** and displayed notably greater body weights from Day 16 to Day 30 **(Figure 5B)**. Moreover, their body weight gain rate was significantly greater from Day 22 to Day 30 **(Figure 5C)**. Body composition analysis via a live body composition analyser revealed increased fat and body fluid contents in the HIF1αKOMBH group **(Figure 5D)**. The levels of energy metabolism and energy efficiency (EE) in the HIF1αKOMBH group were significantly lower than those in the control group **(Figure 5E)**. HE staining of epididymal fat tissue revealed larger, vacuolated fat cells with increased lipid droplets in the HIF1αKOMBH group **(Figure 5G)**, whereas the control group displayed smaller, densely arranged adipocytes **(Figure 5F)**. Adipocyte area analysis via ImageJ revealed significantly larger adipocyte areas in the HIF1αKOMBH group than in the control group **(Figure 5H)**. Similar findings were observed in brown adipose tissue from the scapular region. The HIF1αKOMBH group presented looser adipose tissue with enlarged cells, reduced cytoplasmic content, and larger lipid droplets **(Figure 5I-5K)**.

### Ksr2 was regulated by HIF-1α in the hypothalamus of HFD-fed mice

Compared with that in the control group, the expression of HIF-1α mRNA in the hypothalamus of HIF-1α^flox/flox^ mice injected with AAV-hSyn-cre-GFP was significantly downregulated by approximately 52% (P < 0.05). Additionally, knocking out HIF-1α in the hypothalamic basal nucleus led to a notable reduction in Ksr2 mRNA expression (P<0.05). However, the expression of genes such as Dynlt3, Zfp770, Hadhb, Mcf2l, and Clip2 did not significantly differ **(Figure 5I)**.

### Novel therapeutic candidates for Ksr2 in obesity-related diseases

**Figure 6:**
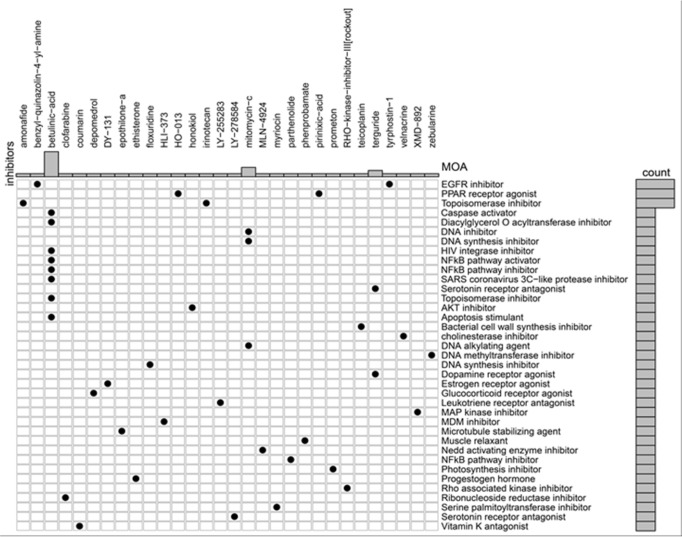
Novel therapeutic targets corresponding to ksr2 genes regulated by HIF-1a in the hypothalamus were predicted via the CMap database. The genes that upregulated ksr2 are shown in the figure.

Our findings highlight Ksr2 as the sole gene modulated by HIF-1α in the hypothalamus. Using the CMAP database to predict drugs targeting Ksr2, more than 2,000 responses were generated. Given the association of Ksr2 with obesity, drugs capable of upregulating its expression are prioritized. The top five candidates identified were Rho-associated kinase inhibitors, amonafide, phenprobamate, irinotecan, and mitomycin-c **(Table 1 and Figure 6)**.

**Table 1:** Small-molecule compounds were identified via CMap analysis to reverse the alterations in differentially expressed genes

Rank	Score	Type	ID	Name	Description
20	73.54	cp	BRD-K23875128	RHO-kinase-inhibitor-III [rockout]	Rho-associated kinase inhibitor
24	72.25	cp	BRD-K56334280	amonafide	Topoisomerase inhibitor
25	70.25	cp	BRD-K22009844	phenprobamate	Muscle relaxant
26	69.77	cp	BRD-K08547377	irinotecan	Topoisomerase inhibitor
30	68.44	cp	BRD-A48237631	mitomycin-c	DNA alkylating agent

## DISCUSSION

In this study, we employed a bioinformatics approach alongside animal models to explore preventive and therapeutic strategies against HFD-induced obesity. Our results highlight the pivotal role of HIF-1 in orchestrating immune cell infiltration in the hypothalamus, particularly in regulating Ksr2 genes linked to HFD-induced hypothalamic inflammation. Based on these findings, we propose five novel therapeutic candidates aimed at modulating the regulated Ksr2 gene to counteract obesity induced by a HFD.

Louveau and cols. (2015) revealed direct connections between lymphatic vessels and the brain’s immune system, underscoring the heightened significance of the immune system within the brain (^36^). A HFD has been found to trigger hypothalamic inflammation, causing neurological alterations and fostering obesity development. The specialized microglia of the brain, which are unique to the central nervous system, can be influenced by interleukins or chemokines from immune cells, thus regulating inflammatory responses in the brain. Our results revealed that memory CD4+ T-cell counts were markedly greater in the HFD-fed samples than in the regular diet-fed samples. Intriguingly, in the mouse weight regain model, obese mice similarly presented increased memory CD4+ T-cell content. Moreover, our study revealed changes in immune cell expression profiles within the HFD group, affecting M0 macrophages, neutrophils, and monocytes.

These findings establish a link between immune cell infiltration and the HIF-1α pathway in the hypothalamus, suggesting potential approaches for addressing HFD-induced obesity. However, recent research highlights the significant role of the microbiota-gut-brain axis in regulating hypothalamic appetite-related neural networks (^37^). Specifically, a HFD elevates endocannabinoid levels, alters the gut microbiota composition, and induces endotoxaemia by increasing lipopolysaccharide (LPS) levels; this triggers cytokine-mediated neuroinflammatory responses by compromising the gut and brain barriers (^38^).

In our animal studies, conditional HIF-1α knockout in the hypothalamus led to obesity even without a HFD. Compared with those in the control group, adipose tissues in the HIF1αKOMBH group presented increased fat content and larger cell size. Additionally, ksr2 gene expression decreased in HIF1αKOMBH mice. KSR2 is an essential intracellular scaffolding protein involved in multiple pathways (^39^). Previous studies have demonstrated that ksr2-/- mice exhibit obesity, elevated insulin levels, and impaired glucose tolerance (^40^-^43^). Furthermore, heterozygous ksr2+/- mice develop obesity when fed a high-fat diet (^40^). These animal models suggest that KSR2 plays a crucial role in energy homeostasis, insulin sensitivity, and cellular fuel oxidation (^40^,^42^).

Further investigations revealed that the function of KSR2 in regulating cellular energy homeostasis is mediated through the activation of 5’-adenosine monophosphate (AMP)-activated protein kinase (AMPK) (^44^). AMPK is a master regulator of cellular energy homeostasis and senses elevated concentrations of AMP and 5’-adenosine diphosphate (ADP), which are indicative of cellular energy depletion (^45^,^46^). By modulating AMPK activity, KSR2 contributes to the maintenance of cellular energy balance and metabolic homeostasis.

These findings suggest that a HFD may initiate hypothalamic inflammation, leading to reduced HIF-1α activity, which subsequently suppresses ksr2 expression, potentially contributing to obesity. Yang and cols. (^47^) reported a 1.82-fold increase in ksr2 expression in channel catfish under hypoxic conditions, suggesting the potential role of HIF-1α in the upregulation of ksr2. While this evidence is not available for mammals, it implies that HIF-1α could regulate ksr2 expression, supporting the idea that ksr2 expression may decrease when HIF-1α is knocked out in mammalian models.

This study suggested that the ratio of memory CD4+ T cells increases after exposure to a HFD. Upon HFD feeding, perivascular macrophages in the arcuate nucleus of the hypothalamus (ARH) express high levels of inducible nitric oxide synthase, leading to the release of a substantial amount of nitric oxide (NO) (^48^). NO can inhibit T-cell apoptosis and stimulate the production of recognition molecules on antigen-presenting cells, such as CD1, a lipid-presenting protein. Notably, the activation of CD1-dependent signalling has been associated with increased body mass gain and the exacerbation of diet-induced hypothalamic inflammation (^49^).

Furthermore, NO stimulates the differentiation and polarization of helper T (Th1) cells through the cyclic guanosine monophosphate (cGMP) pathway (^50^). While Th1 CD4+ T cells are commonly generated in response to both acute and persistent infections, this population can be lost over time following persistent activation (^51^). However, NO may help maintain the Th cell population by preventing their apoptosis.

Another mechanism contributing to T-cell memory formation is the activation of the cyclic adenosine monophosphate (cAMP)-protein kinase A signalling pathway, which prevents T-cell death after activation and enhances the generation of memory T cells (^52^). Interestingly, NO can activate AMP-activated protein kinase (AMPK) by modulating phosphodiesterases (^53^), which can also modulate the cAMP and cGMP signalling pathways. Given that the kinase suppressor of Ras 2 (KSR2) affects the activity of AMPK, it is plausible that KSR2 may also influence the formation of memory CD4+ T cells. This potential link could be mediated through the modulation of AMPK activity, which in turn affects the cross-talk between the cAMP and cGMP signalling pathways, ultimately impacting T-cell survival and memory formation.

This study, via the CMAP database, identified the top candidates (Rho-associated kinase inhibitors, amonafide, phenprobamate, irinotecan, and mitomycin-C) to increase ksr2 gene activity. Phenprobamate is a muscle relaxant, while amonafide inhibits topoisomerase I, and irinotecan inhibits topoisomerase II. Mitomycin-C, a DNA alkylating agent, weakens the compact structure of DNA, potentially increasing gene expression by facilitating interactions with the translation machinery. Ksr2 methylation, which is linked to rectal adenocarcinoma survival (^54^), reveals the role of DNA methylation in compacting DNA and silencing genes, contrasting alkylation effects. These findings underscore the vital epigenetic role of ksr2 in cellular function.

Amonafide and irinotecan, which are potent anticancer drugs, effectively inhibit topoisomerase activity and have been utilized for decades. These drugs inhibit cell proliferation by trapping replication or transcription machinery (^55^) and induce apoptosis through a ROS-dependent DNA damage signalling pathway (^56^,^57^). Camptothecin, a topoisomerase inhibitor, effectively treats obesity in mice by activating glial-derived neurotrophic factor (GDNF) receptor alpha-like (GFRAL) (^58^). The therapeutic impact of camptothecin mimicked the function of the ksr2 gene, suggesting that similar topoisomerase inhibitors may also prevent obesity.

Stress triggers a complex “fight-or-flight” reaction, releasing hormones such as cortisol into the body (^59^,^60^). Prolonged stress can cause muscle tension, resulting in stiffness and discomfort. Muscle relaxants can alleviate this effect by easing contraction. Stress is associated with obesity through various cognitive, behavioural, and physiological mechanisms (^61^). Stress can impair self-regulation and drive excessive intake of calorie-dense foods; it also triggers the release of hormones such as ghrelin, leptin, and neuropeptide Y, influencing eating behaviour (^62^-^64^). Hydralazine, a muscle relaxant, has been shown to reduce body fat by enhancing abdominal subcutaneous adipose tissue lipolysis in both animals and humans (^65^). These findings suggest that phenprobamate may have a similar effect on weight control and ksr2 stimulation.

Rho-associated kinase inhibitors have emerged as potential treatments for metabolic conditions such as obesity, insulin resistance, dyslipidaemia, and hypertension (^66^-^70^). Animal studies suggest that Rho kinase inhibitors positively impact body weight regulation and adipose tissue metabolism (^71^,^72^). These inhibitors show promise in reducing food intake, increasing energy expenditure, and impeding fat accumulation and adipocyte differentiation.

While there are limited direct reports confirming the impact of these drugs on obesity, our findings suggest a potential effect of these drugs. Additional animal or randomized clinical studies are crucial to validate or refine our observations.

In conclusion, using a bioinformatics approach alongside animal models, we identified the critical role of HIF-1 in immune cell infiltration within the hypothalamus of HFD-fed mice, linking HFD-induced inflammation to the regulation of the Ksr2 gene through HIF-1. Furthermore, we suggest five potential drugs to target the regulated Ksr2 gene and counter HFD-induced obesity, pending in vitro and in vivo validation for further research.
